# *Mycobacterium nebraskense* Isolated from Patients in Connecticut and Oregon, USA

**DOI:** 10.3201/eid3103.240608

**Published:** 2025-03

**Authors:** Mark L. Metersky, Ashley J. Losier, David A. Fraulino, Theodore A. Warnock, Cara D. Varley, Angela M. Le, Kevin L. Winthrop, John R. McArdle, Salika M. Shakir, Reeti Khare

**Affiliations:** University of Connecticut School of Medicine, Farmington, Connecticut, USA (M.L. Metersky, D.A. Fraulino, J.R. McArdle); Yale University School of Medicine, New Haven, Connecticut, USA (A.J. Losier); Oregon Health and Science University, Portland, Oregon, USA (T.A. Warnock, C.D. Varley, A.M. Le, K.L. Winthrop); Portland State University, Portland (T.A. Warnock, C.D. Varley, K.L. Winthrop); University of Utah ARUP Laboratories, Salt Lake City, Utah, USA (S.M. Shakir); National Jewish Health, Denver, Colorado, USA (R. Khare)

**Keywords:** *Mycobacterium nebraskense*, nontuberculous mycobacteria, bronchiectasis, bacteria, respiratory infections, Connecticut, Oregon, United States

## Abstract

*Mycobacterium nebraskense* infection is rarely encountered; only 7 human cases have been reported worldwide since the initial report of 5 cases in Nebraska, USA, in 2004. We report 9 patients from Connecticut and 2 from Oregon, USA, who had *M. nebraskense* isolated from respiratory secretions; 7 patients met the American Thoracic Society/Infectious Diseases Society of America criteria for nontuberculous mycobacterial pulmonary disease. In 4 cases, the organism was isolated 1 time and caused brief or no symptoms. Most cases in Connecticut were reported after 2017. Antimicrobial drug susceptibility testing of 6 isolates showed clarithromycin susceptibility. In 2 cases, infection was refractory to treatment. The 9 Connecticut patients lived in 8 different towns; thus, a common water supply did not explain the high frequency of *M. nebraskense* isolation. *M. nebraskense* is a clinically significant cause of nontuberculous mycobacterial pulmonary disease in Connecticut; continued surveillance will be needed to determine its frequency and optimum treatment.

*Mycobacterium nebraskense* is a slow-growing, scotochromogenic mycobacterium first described in 2004 after it was isolated from 5 patients at the University of Nebraska Medical Center in Omaha, Nebraska, USA (*1*). Each patient had symptomatic lung disease; more detailed clinical information was reported for 1 patient with underlying emphysema, bronchiectasis, and a mass-like lung lesion who was thought to have nontuberculous mycobacterial pulmonary disease (NTM-PD) caused by *M. nebraskense* (*2*). Since those initial descriptions, only 7 additional isolates from humans have been reported (*3*); 6 were from different states within the United States, and 1 was from Japan. *M*. *nebraskense* has rarely been isolated from skin lesions in animals (*4*) or from nonpotable water reservoirs (*5*). 

We report 11 patients from Connecticut and Oregon, USA, who had *M. nebraskense* isolated from respiratory samples. This previously rare organism has recently been the third most common nontuberculous mycobacteria (NTM) isolated from patients receiving treatment in a dedicated bronchiectasis center at the University of Connecticut Health Center (after *M. avium* complex [MAC] and *M. gordonae*). The University of Connecticut Health Institutional Review Board (IRB) determined this work was exempt from full IRB review (IRB no. 23X-084-02); the study was also approved by the Oregon Health and Science University IRB (approval no. IRB00003522).

## Methods

The Centers for Disease Control and Prevention (Atlanta, GA, USA) identified the Connecticut case (CT-C) 1 isolate by using 16S rRNA gene sequencing (no further details available). ARUP Laboratories (Salt Lake City, UT, USA) identified isolates CT-C2 and CT-C4 by using 16S rRNA Sanger gene sequencing. The first 500 bp of the 16S rRNA gene was sequenced by using 5F-T and 534R-T primers and analyzed by using the RipSeq database (Pathogenomix, https://www.pathogenomix.com). Results were reported in accordance with Clinical and Laboratory Standards Institute criteria for identifying mycobacteria at the species level (*6*) if the sequence was 100% identical. ARUP Laboratories identified 6 CT-C3 respiratory isolates by using either 16S rRNA gene sequencing or matrix-assisted laser desorption/ionization time-of-flight mass spectrometry (MALDI Biotyper; Bruker Daltonics, https://www.bruker.com); a score of >1.9 was required for positive species level identification. QUEST Laboratories (Secaucus, NJ, USA) identified the CT-C5 isolate by using DNA sequencing with an *M. nebraskense*–specific probe (no further information was available). The Yale New Haven Hospital microbiology laboratory (New Haven, CT, USA) identified CT-C7 and CT-C8 isolates by using 16S rRNA gene sequencing. ARUP Laboratories identified the CT-C9 isolate by using mass spectrometry as described previously. The National Jewish Hospital Advanced Diagnostic Laboratories (Denver, CO, USA) identified the Oregon case (OR-C1 and OR-C2) isolates by using *rpoB* gene Sanger sequencing (732-bp region). Isolates from Oregon were identified by comparing results to sequences in GenBank, requiring a >97% match.

## Cases

### CT-C1

We evaluated a 61-year-old man in 2008 for productive cough and hemoptysis, as previously described (*7*). He had a history of chronic obstructive pulmonary disease, cardiomyopathy, hypertension, type 2 diabetes, and smoking. His physical examination was unremarkable. A chest computed tomography (CT) scan revealed no infiltrates or bronchiectasis. Pulmonary function tests showed mild to moderate obstruction. Bronchoalveolar lavage (BAL) cultures grew *Aspergillus fumigatus*, *Escherichia coli*, and NTM most consistent with MAC according to high-performance liquid chromatography (HPLC). We did not treat the patient because the CT scan had not revealed bronchiectasis or nodules consistent with NTM-PD.

Fevers, sweats, and increased sputum production developed ≈6 months later. Another chest CT scan revealed ill-defined nodules of various sizes throughout both lungs. After another sputum sample was obtained, we treated the patient with rifampin, ethambutol, and azithromycin for a presumed MAC pulmonary infection. A commercial laboratory using an unknown method identified the organism as *M. interjectum*. Because of uncertainty regarding the identity of the organism, we sent the BAL isolate to the Centers for Disease Control and Prevention; HPLC revealed an organism consistent with *M. scrofulaceum*, but 16S rRNA sequencing identified *M. nebraskense*. The patient’s symptoms rapidly improved, a sputum sample obtained 1 month later was acid-fast bacillus (AFB) negative, and a repeat chest CT scan showed near complete resolution of nodules. After ≈2 months, we discontinued ethambutol, and he remained on rifampin and azithromycin for another 10 months.

The patient required bronchial stenting because of stenosis of his left mainstem bronchus, thought to be a result of an endobronchial NTM infection. Samples from 2 bronchoscopies performed after 6 and 7 months of therapy did not grow mycobacteria. An organism consistent with *M. scrofulaceum* (according to HPLC) was cultured from a sputum sample obtained when he had completed ≈1 year of therapy; however, no disease was evident, and we did not reinstitute therapy. Subsequent sputum cultures did not grow mycobacteria, and a chest CT scan performed ≈6 months after therapy completion showed no disease. He remained well for 14 months after completing therapy and was then lost to follow-up.

### CT-C2

**Figure 1:**
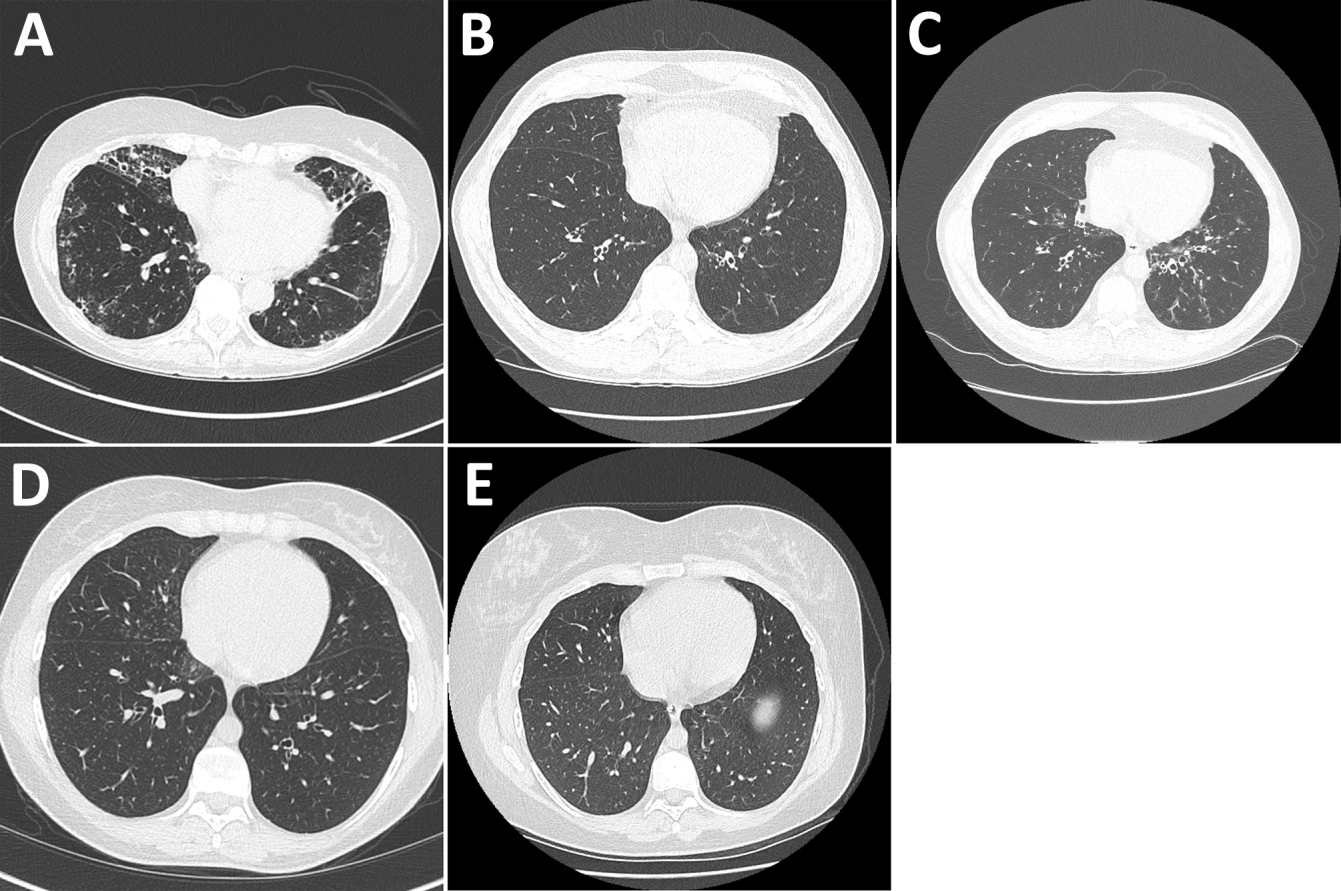
High-resolution chest computed tomography scans from study of *Mycobacterium nebraskense* isolated from patients in Connecticut and Oregon, USA. A) Scan from case 2 in Connecticut showing bronchiectasis in the right middle and lower lobe, left lower lobe, and lingula and tree-in-bud nodularity in the lingula and right and left lower lobes of the lung. B) Scan slice from case 3 in Connecticut through the lower lung lobes showing 1 borderline dilated airway in the left lower lobe, performed in May 2019. C) Scan slice from case 3 at the same anatomic level showing numerous dilated airways and airway wall thickening in the left lower lobe of lung, performed in April 2020 soon after the first isolation of *M. nebraskense*. D) Scan from case 4 in Connecticut showing tree-in-bud nodularity in the right middle and lower lobes of the lung when patient had *M. nebraskense* isolated from her sputum in August 2020. E) Scan from case 4 in Connecticut showing resolution of the tree-in-bud nodularity in the right middle and lower lobes of the lung, performed in December 2022.

We evaluated a 69-year-old woman in 2010 for bronchiectasis. Initial sputum mycobacterial cultures were negative; however, MAC grew from 2 sputum sample cultures in 2011. Her chest CT scan showed bronchiectasis and scattered tree-in-bud nodularity (Figure 1, panel A). Because she was doing well clinically, she continued only on an airway clearance regimen. Numerous sputum mycobacterial cultures were negative until 2018, when a sample grew *M. nebraskense*, identified by 16S rRNA gene sequencing. A repeat CT scan showed progression of her tree-in-bud nodularity and bronchiectasis when compared with a 2012 scan. Four sputum mycobacterial cultures during 2019–2022 were negative except for 1 isolation of *M. gordonae*. In January 2023, she reported daily productive cough. She had ovarian cancer and was receiving chemotherapy. She was not regularly performing airway clearance. A sputum culture from January 2023 grew *M. nebraskense*. Antimicrobial susceptibility testing could not be completed because of poor organism growth after using the CLSI approved test method for slow growing mycobacteria. We did not start her on antimycobacterial drug therapy because her symptoms were well controlled with airway clearance, and her CT scan findings had not progressed. She died from ovarian cancer in September 2023.

### CT-C3

We saw a 55-year old man who had asthma since childhood. He had a 30 pack-year (1 pack/day for 30 years) history of cigarette smoking but had quit for several years. Cough and congestion persisted despite aggressive asthma treatment. Mycobacterial cultures were negative in 2017, 2018, and 2019. In 2020, two sputum cultures grew *M. nebraskense*; drug susceptibility testing could not be done because of poor organism growth. A chest CT scan in 2020 revealed tree-in-bud nodularity, bronchiectasis, and mucus plugging, most prominent in the left lower lobe; most findings were new compared with a 2019 CT scan (Figure 1, panels B, C). We started him on azithromycin, ethambutol, and rifampin. After 12 months of therapy, despite clinical and radiographic improvement, he remained sputum-culture positive. After ≈18 months of therapy, his sputum AFB cultures were negative. Three months later, he had reemergence of cough, and sputum cultures again grew *M. nebraskense*. Drug susceptibility testing revealed susceptibility to all agents tested (Table 1). We treated him with azithromycin, rifampin, ethambutol, and linezolid. We discontinued all therapy in February 2024 because of persistent culture positivity with an acceptable symptom burden. A direct smear of biofilm from a water filter in his house was strongly positive for AFB and grew *M. nebraskense*.

**Table 1:** Antimicrobial drug susceptibility testing results for 6 *Mycobacterium nebraskense* isolates from patients in Connecticut and Oregon, USA*

Drug	MIC, μg/mL					
	CT-C3	CT-C5	CT-C6	OR-C1	OR-C2, pretreatment	OR-C2, during treatment
TMP/SMX	<0.25/4.75, S	4/76, R	S	NA	NA	NA
Doxycycline	<0.12, S	NA	NA	NA	NA	NA
Linezolid	<1, S	2, S	NA	2.0, S	NA	64.0, R
Rifabutin	<0.12, S	0.25, S	S	<0.25, S	>0.5, R	>8.0, R
Amikacin	<1, S	2, S	8, S	4.0, I	4.0, I	16.0, R
Moxifloxacin	0.03, S	0.03, S	S	0.25, S	<0.5, S	16.0, S
Ciprofloxacin	<0.12, S	1, S	S	0.5, S	2.0, I	8.0, R
Clarithromycin	<0.06, S	0.12, S	0.5, S	0.12, S	<4.0, S	8.0, S
Minocycline	0.5, S	0.03, S	NA	NA	NA	NA
Rifampin	0.12, S	1, S	S	0.5, S	1.0, S	16.0, R

*CT-C, Connecticut-case; I, intermediate; NA, not applicable; OR-C, Oregon-case; R, resistant; S, susceptible; TMP/SMX, trimethoprim-sulfamethoxazole.

### CT-C4

We saw a 19-year-old woman in 2015 with a history of primary ciliary dyskinesia diagnosed at 4 years of age, sinusitis, otitis media, and recurrent *Stenotrophomonas maltophilia* respiratory infections. Her treatment included prophylactic trimethoprim/sulfamethoxazole and airway clearance with a hand-held positive pressure oscillatory device. She had done well for several years, having minimal daily cough and sputum production. A CT scan from 2 years earlier was unremarkable. In 2020, she began having intermittent low-grade fevers and increased cough and sputum production, which persisted for several months. A CT scan showed new areas of tree-in-bud nodularity in the right middle and lower lobes (Figure 1, panel D). A sputum mycobacterial culture from August 2020 grew *M. nebraskense*, identified by partial 16S rRNA gene sequencing. Antimicrobial susceptibility testing could not be completed because of poor organism growth. A sputum bacterial culture revealed normal oral flora. Her symptoms persisted; a repeat mycobacterial culture from December 2020 was negative and, over several months, her fevers and cough improved. A repeat CT scan in 2022 showed resolution of the tree-in-bud nodularity in the right middle and lower lobes (Figure 1, panel E), and she has done well since then.

### CT-C5

A 64-year-old woman was referred to us for evaluation of asthma in August 2021. She reported a history of pneumonia and asthma diagnosed at age 7. She had been noticing shortness of breath with exertion during the previous 2–3 years. She reported a chronic cough with sputum production. She had smoked 6 cigarettes per day when she was 16–25 years of age. She had lost ≈10 pounds in body weight during the preceding 2 years and had a body mass index of 16.5 kg/m^3^. Medications included inhaled bronchodilators and, for the previous 9 months, inhaled corticosteroids. Spirometry revealed severe obstruction; her oxygen saturation was 86% on room air. A chest CT scan revealed diffuse cylindrical bronchiectasis with marked bronchial wall thickening, multifocal mucoid impaction, and tree-in-bud nodularity. Laboratory evaluation showed no underlying cause for her bronchiectasis. Her sputum culture was positive for multiple gram-negative bacilli, including *Alcaligenes faecalis* and *Pseudomonas aeruginosa.* We treated her with levofloxacin, and she had transient improvement in sputum production.

In February, 2 sputum mycobacterial cultures grew *M. nebraskense*, and drug susceptibility testing was performed (Table 1). Percussion vest therapy was initiated. A repeat sputum bacterial culture was positive for *P. aeruginosa.* We started her on nebulized tobramycin, azithromycin, ethambutol, and rifampin. She was hospitalized for acute respiratory failure, and we treated her with intravenous cefepime in addition to her previous regimen; she showed modest improvement and was discharged on oxygen. She returned to the hospital because of worsening respiratory failure, elected comfort measures, and died 6 weeks after initiating antimycobacterial therapy.

### CT-C6

We saw a 44-year-old woman for dyspnea and chronic cough producing yellow sputum in September 2022. A CT scan revealed bilateral interstitial lung disease but no bronchiectasis or nodules. Sputum mycobacterial culture revealed MAC growth. A second mycobacterial culture a month later was negative, whereas a third culture in November 2022 revealed *M. nebraskense* according to DNA sequencing; drug susceptibility testing was performed (Table 1). Further evaluation showed an elevated antinuclear antibody titer of 1:1,250 with a centromere pattern and elevated RNA polymerase III antibody, leading to a presumptive diagnosis of systemic sclerosis. Three subsequent sputum mycobacterial cultures 2, 3, and 6 months later were negative. Her cough persisted but has been less productive.

### CT-C7

A 73-year-old woman sought care in August 2020 for recurrent pneumonia, chronic cough with yellow sputum, fatigue, fevers, and weight loss. She had end-stage renal disease secondary to membranoproliferative nephritis status postkidney transplant and was taking prednisone. Results of pulmonary function testing were unremarkable. Her chest CT scan demonstrated bilateral bronchiectasis, tree-in-bud nodules, and mucus plugging. Testing for underlying etiologies of bronchiectasis was unrevealing. We thought that her bronchiectasis was secondary to recurrent chest infections in an immunocompromised host. We recommended airway clearance therapy. A sputum culture grew *Haemophilus parainfluenzae*; a negative mycobacterial culture was obtained at the time she sought care. During the following year, she had several bronchiectasis exacerbations requiring antimicrobial drugs for coverage of *Staphylococcus aureus*, *P. aeruginosa*, and isolated *M. nebraskense* from sputum samples obtained in November 2021 and April 2022. No drug susceptibility testing was available. We observed progression of bronchiectasis on repeat chest CT imaging. We started her on nebulized tobramycin solution because of frequent bronchiectasis exacerbations and a persistent productive cough. She noted substantial improvement in cough and fatigue, and her body weight stabilized after using an augmented airway clearance regimen and nebulized tobramycin. She has remained without treatment for *M. nebraskense* infection because of stable symptoms.

### CT-C8

In March 2021, a 69-year-old man with a history of coronary artery disease, chronic lymphocytic leukemia (treated with ibrutinib), gastroesophageal reflux, diabetes, and allergic rhinitis sought care for chronic productive cough and chest imaging demonstrating bronchiectasis. His physical examination and spirometry results were unremarkable. Evaluation for underlying causes of bronchiectasis was unrevealing. We started him on airway clearance with a hand-held positive expiratory pressure oscillatory device. The lung CT scan showed chronic scattered nodules (<6 mm), mucus impaction, and mild bronchiectasis in the right lower lobe. His cough continued, and a sputum culture in December 2021 grew methicillin-resistant *S*. *aureus*, *A. fumigatus*, and *M. nebraskense*; drug susceptibility testing was not available. In February 2022, he was hospitalized with *Pneumocystis jirovecii* pneumonia. A mycobacterial culture of a BAL sample was negative. He improved after treatment for pneumonia and remains with mild dyspnea and chronic cough; *M. nebraskense* has not been isolated from subsequent sputum mycobacterial cultures.

### CT-C9

We evaluated a 56-year-old woman in April 2023 for cough producing scant sputum, chest pain, and an abnormal CT scan. She had no remarkable medical history. A chest CT scan in February 2023 revealed multifocal patchy consolidation, several areas of tree-in-bud nodularity, and bronchiectasis, most prominent in the right middle and upper lobes and lingula. Pulmonary function testing was unremarkable. A repeat CT scan showed partial improvement of the patchy consolidation and tree-in-bud nodularity. One of 3 sputum mycobacterial cultures from May 2023 grew *M. nebraskense.* Susceptibility testing could not be done by the reference laboratory because of poor organism growth. A respiratory bacterial culture was negative. Evaluation for underlying causes of bronchiectasis was unrevealing. We did not provide specific treatment. By December 2023, her cough had resolved, although a CT scan revealed only partial improvement of some of the tree-in-bud nodularity observed in April. Bronchoscopy was performed in January 2024; a BAL sample was negative for mycobacteria.

### OR-C1

In 2013, we evaluated an 80-year-old woman who was born and raised in Oregon for cough that had been present for ≈10 years and for more recent cryptogenic organizing pneumonia, shortness of breath, low-grade fevers, and increased sputum production. She had gastroesophageal reflux and allergies managed by tap water sinus rinses. We obtained 3 sputum mycobacterial cultures; 1 grew *M. lentiflavum*, identified by gene sequencing. Tap water sinus rinses were discontinued, and we started her on daily azithromycin, ethambutol, and rifampin; culture conversion occurred after 5 months of therapy. We performed BAL because of minimal radiologic improvement in the lungs, which yielded negative fungal, bacterial, and mycobacterial cultures. Areas of radiographic progression were more consistent with cryptogenic organizing pneumonia; improvements in imaging findings were more consistent with NTM-PD. We discontinued treatment after 1 year, and within 3 months, her cough and sputum production returned.

In 2015, two sputum mycobacterial cultures grew *M. nebraskense*, which was resistant to ethambutol (but susceptible to rifampin/ethambutol combination), and 1 culture was positive for *M. scrofulaceum*. We resumed daily azithromycin, ethambutol, and rifampin in mid-2016 and observed serial negative mycobacterial cultures. We changed her regimen to azithromycin and rifampin; she completed 1 year of therapy and had radiologic and symptomatic improvement. Surveillance cultures remained negative until 2018, when MAC was identified in 2 sputum samples. Radiologic progression of disease and continued productive cough warranted resumption of her antimicrobial drug regimen in early 2019, completed in late 2021.

### OR-C2

A 71-year-old woman, originally from Vietnam, who had a history of colon cancer status postcolectomy and diabetes mellitus had widespread bronchiectasis with tree-in-bud infiltrates that was discovered incidentally on lung CT scan as part of her malignancy evaluation. Mild progression of bronchiectasis was observed mostly in the right middle lobe and lingula on a subsequent CT scan 2 months later, prompting a bronchoscopy in 2014. Cultures revealed mucoid *P. aeruginosa* and were 1+ AFB smear positive and grew *M. nebraskense*, identified by *rpoB* gene sequencing. We began daily rifampin, ethambutol, and azithromycin treatment. After 11 months, another sputum sample was 2+ AFB smear positive and grew *M. nebraskense* in culture; antimicrobial susceptibility testing showed newly acquired resistance to ethambutol and rifampin, separately or in combination, and macrolide susceptibility (Table 1). We replaced ethambutol with moxifloxacin and referred her for surgical consultation for possible resection of severe bronchiectatic regions and for an enlarging ground-glass nodule in the right lower lung lobe, possibly malignant. After continued culture positivity, despite 21 months of therapy, and the development of antimicrobial drug resistance, we placed her on intravenous amikacin for 2 weeks before and after a right middle lobectomy and right lower lobe wedge resection in 2016. Histopathology confirmed stage IA pulmonary adenocarcinoma; mycobacterial cultures were negative. We discontinued treatment after 25 months. She subsequently required treatment for frequent exacerbations related to *P. aeruginosa* and NTM-PD caused by *M. paraffinicum* and MAC.

## Results

We summarized the clinical characteristics of each case (Table 2). Eight (73%) of 11 patients were female, 3 (27%) male. Underlying bronchiectasis was present in 6 (55%) patients; 7 (64%) patients met the American Thoracic Society/Infectious Disease Society of America criteria for NTM-PD. Initial antimicrobial susceptibility testing of 6 isolates revealed uniform macrolide susceptibility (Table 1). The 2 isolates from Oregon had intermediate sensitivity to amikacin, and 1 of those had in vitro resistance to rifampin. Five (45%) patients received antimycobacterial drug treatment; 2 required prolonged therapy because of rapidly recurrent disease after initial completion of treatment for 18 and 21 months.

**Table 2:** Summary of 11 cases of *Mycobacterium nebraskense* infection in Connecticut and Oregon, USA*

Case no.	Year†	Age, y/sex	Underlying illnesses	Immunosuppressed	Met criteria for pulmonary disease‡	Antimycobacterial treatment	Outcome
CT-C1	2008	61/M	Chronic obstructive pulmonary disease	No	Yes, assuming second *M. scrofulaceum* identification was *M. nebraskense*	Yes	Prolonged culture conversion
CT-C2	2018	69/F	Bronchiectasis at initial isolation, ovarian cancer diagnosed in 2019	No initially, then prolonged chemotherapy	Yes	No	Died from ovarian cancer in 2023
CT-C3	2020	57/M	Asthma	No	Yes	Yes	Persistent infection despite prolonged treatment
CT-C4	2020	24/F	Primary ciliary dyskinesia	No	No, characteristic CT findings but only 1 positive culture	No	Spontaneous resolution of symptoms and CT findings, subsequent negative cultures
CT-C5	2022	64/F	Asthma, bronchiectasis	No	Yes	Yes	Died from respiratory failure 6 weeks after initiating treatment
CT-C6	2022	44/F	Interstitial lung disease from systemic sclerosis	No	No	No	Spontaneous culture conversion
CT-C7	2021	71/F	Kidney transplantation	Yes	Yes	No	Symptom control with airway clearance alone
CT-C8	2021	69/M	Chronic lymphocytic leukemia	Yes	No	No	Spontaneous culture conversion
CT-C9	2023	56/F	None	No	No	No	Spontaneous resolution of symptoms and some CT findings, subsequent negative cultures
OR-C1	2015	82/F	Gastroesophageal reflux, hiatal hernia, allergic rhinitis	No	Yes	Yes	Culture conversion during treatment
OR-C2	2014	71/F	Bronchiectasis, type 2 diabetes, colon cancer	No	Yes	Yes	Converted after prolonged therapy, including surgical resection
Overall	NA	60.7 ±15.7 (mean ±SD), 8/11 (73%) female	NA	2/11 (18%) immunosuppressed at initial *M. nebraskenske* isolation	7/11 (64%) met criteria for NTM-PD	5/11 (45%) received antimycobacterial therapy	NA

*Mycobacterium nebraskense was isolated from respiratory samples. CT-C, Connecticut-case; NA, not applicable; NTM, nontuberculous mycobacteria; NTM-PD, nontuberculous mycobacterial pulmonary disease; OR-C, Oregon-case. †Year *M. nebraskense* was first isolated. ‡Met the American Thoracic Society/Infectious Disease Society of America criteria for NTM-PD by having >2 positive cultures and characteristic CT imaging showing bronchiectasis or tree-in-bud nodularity.

**Figure 2:**
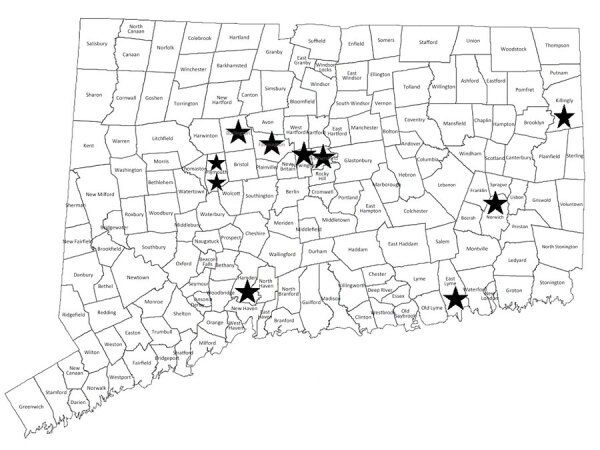
Towns of residence (stars) for 9 patients in Connecticut, USA, who had *Mycobacterium nebraskense* isolated during 2008–2023.

Most Connecticut patients lived in various locales (Figure 2); thus, they did not all share the same household water supply. Two patients lived in the same small town (population ≈5,000), suggesting a possible common environmental source of mycobacteria. None of the 11 patients had medical visits on the same day, making nosocomial patient-to-patient transmission unlikely.

*M. nebraskense* isolates were processed in several different laboratories over several years and thus, a pseudo-outbreak related to contaminated laboratory reagents was unlikely. However, we performed mycobacterial cultures on reagents potentially subject to contamination at the University of Connecticut Health Center mycobacteriology laboratory, where most isolates in this series were processed: NAC-PAC NALC tablets, NPC-67 neutralizing buffer, and PRB pellet resuspension buffer (Alpha-Tec Systems, https://www.alphatecsystems.com); antibiotic mixture consisting of polymyxin B, trimethoprim, amphotericin B, azlocillin, nalidixic acid; and oleic albumin dextrose catalase growth supplement. All cultures were negative.

## Discussion

*M. nebraskense* infections were first described in 2004 in Nebraska (*1*), and only 7 additional cases have been reported worldwide since then. We report a series of 11 patients who had *M. nebraskense* isolated from respiratory samples; 9 were from Connecticut. Seven cases met the criteria for NTM-PD. Two patients remained refractory to treatment with 4 antimycobacterial drugs for extended periods of time. Two patients (CT-C4 and CT-C9) who had subacute symptoms and a CT scan suggestive of NTM-PD cleared their sputum and had improvement of their symptoms and CT findings without treatment, suggesting that *M. nebraskense* might cause self-limiting infection and symptoms, similar to a previously reported case (*8*).We observed a wide range of clinical manifestations in our case series, including patients with a single isolate and no obvious clinical sequela and those with progressive bronchiectasis and treatment-refractory disease. Temporal association with progressive bronchiectasis (CT-C3) and bronchial stenosis (CT-C1) suggests that *M. nebraskense* can directly damage large airways. CT scan results were also variable and included bronchiectasis, tree-in-bud nodularity, and large nodules.

Although most patients did not have systemic immunosuppression, immunocompetent patients had conditions thought to increase the risk for NTM-PD, such as bronchiectasis (*9*), gastroesophageal reflux (*10*), and inhaled corticosteroid use (*11*). Those factors likely increased risk for NTM-PD by impairing local airway host defenses against infection (*12*) and in the case of gastroesophageal reflux, by increasing airway exposure to NTM.

Despite the pathogenicity of *M. nebraskense* in several patients, others had only a single positive culture and no persistent symptoms. This result demonstrates that initial isolation of this organism does not always indicate the presence of NTM-PD and should generally not prompt antimycobacterial therapy, similar to other NTM such as MAC. Serial mycobacterial cultures and careful clinical assessment and follow-up are indicated before deciding if treatment is warranted.

Drug susceptibility testing revealed uniform in vitro susceptibility to clarithromycin, similar to prior case reports (*3*), but some isolates were resistant to other first-line NTM drugs, including rifamycins and amikacin. In 3 cases, the reference laboratory was not able to perform susceptibility testing because of poor growth of the organism after identification.

In conclusion, *M. nebraskense* has only been reported in rare single case reports, except for the initial report in 2004 from Nebraska (*1*). We report that this organism can be a clinically critical pathogen in Connecticut and has caused sporadic disease in at least 1 other US state. It is unclear whether the increased isolation frequency of this organism in Connecticut represents a true increase in prevalence or is a result of increased availability of molecular methods to identify NTM. Increased research on NTM-PD will be needed to improve diagnosis and treatment of *M. nebraskense* and other NTM infections.

### Addendum

Since submission of this manuscript, 2 more patients from Connecticut have had *M*. *nebraskense* isolated from their sputum, 1 at Yale University (isolated 1×) and 1 at University of Connecticut (isolated 2×) (A.J. Losier, M.L. Metersky, unpub. data). Including those 2 patients, *M*. *nebraskense* has now been isolated from a total of 11 patients from Connecticut, 10 since 2018.
